# Pulses and Cancer Outcomes: A Scoping Review of Human Studies on Risk Reduction

**DOI:** 10.3390/nu18132064

**Published:** 2026-06-24

**Authors:** Mohd Naeem Mohd Nawi, Nurliayana Ibrahim, Tay Bee Yong, Aswir Abd Rashed, Vimala Rmt Balasubramaniam

**Affiliations:** 1 Nutrition Unit, Nutrition, Metabolism and Cardiovascular Research Centre, Institute for Medical Research, National Institutes of Health, Ministry of Health, Setia Alam, Shah Alam 40170, Selangor, Malaysia; liayana.psh@moh.gov.my (N.I.); aswir@moh.gov.my (A.A.R.); vimala.rmt@moh.gov.my (V.R.B.); 2 Bacteriology Unit, Infectious Disease Research Centre, Institute for Medical Research, National Institutes of Health, Ministry of Health, Setia Alam, Shah Alam 40170, Selangor, Malaysia; tayby@moh.gov.my

**Keywords:** pulses, cancer, risk, mechanistic

## Abstract

**Background/Objectives**: Pulses are nutrient-dense, low-glycaemic legumes rich in fibre and bioactive compounds that may modulate carcinogenesis through effects on diet quality, metabolism, and the gut microbiome. This scoping review mapped human evidence on pulses in relation to cancer risk reduction and related mechanistic and survivorship-relevant outcomes. **Methods**: Following the Preferred Reporting Items for Systematic reviews and Meta-Analyses extension for Scoping Reviews (PRISMA-ScR) and Joanna Briggs Institute (JBI) Population, Concept and Context (PCC) guidance, we searched CENTRAL, Scopus and PubMed (2014–31 December 2025), supplemented by backward and forward citation tracking, for English-language human studies in which pulses were a defined exposure or intervention and cancer-specific clinical outcomes or biomarkers were reported. Exposures are described using the original ‘legume’ terminology, with pulse-specific interpretation restricted to FAO-defined pulses or clearly dry pulse forms and to pulse-dominant legume intake where the constituent items were predominantly pulses but preparation was not specified. **Results**: After screening 1244 records, 15 studies met the inclusion criteria, comprising five case–control studies, five 4-week randomised controlled trials (RCTs), one 8-week randomised crossover trial, one controlled feeding study, two prospective cohort studies, and one other prospective study. Observational data from a single pooled case–control study suggest that higher pulse-dominant legume intake is compatible with modestly lower colorectal cancer risk, although the findings are mixed and often attenuate after adjustment for lifestyle and dietary confounders. Evidence for breast and oesophageal cancer and all-cancer mortality is limited, frequently subgroup-specific or highly sensitive to confounder control, and survivorship endpoints are represented mainly by short-term mechanistic and feasibility trials in colorectal cancer survivors rather than by long-term clinical outcomes. Notably, five of these navy bean interventions were conducted by a single research group using similar protocols, which constrains the independence of replication. **Conclusions**: Pulses can be considered practical components of cancer-protective dietary patterns, especially for colorectal cancer, but the heterogeneity of study designs, short-term interventions, limited sample sizes, and lack of preparation-specific exposure data preclude firm causal inferences; longer-term, rigorously designed trials and detailed observational work are needed to refine pulse-based recommendations for cancer risk reduction and to clarify any role in survivorship care.

## 1. Introduction

Pulses are the dry, edible seeds of leguminous plants in the *Fabaceae* family, harvested at maturity for use in human diets and animal feed. According to the Food and Agriculture Organization (FAO) of the United Nations, pulses are defined as leguminous crops harvested solely as dry seeds, excluding legumes grown primarily for oil (such as soybeans and groundnuts), fresh vegetable legumes (such as green peas and green beans), and legumes used exclusively for forage or soil improvement (such as clover or alfalfa) [1]. In this FAO-based classification, pulses represent a subgroup of legumes characterised by their low oil, high protein and high fibre contents. Within this framework, FAO classifies pulses into 11 main categories: lentils, chickpeas, vetches, dry peas, Bambara beans, lupins, cowpeas, dry beans, pigeon peas, and dry broad beans and “pulses not elsewhere specified,” the latter grouping minor pulse species that do not fall within the preceding categories [2]. These categories include widely consumed species such as lentils, black-eyed peas, pinto beans, chickpeas, navy beans, split peas, kidney beans, and black beans [1,3,4]. Each varies in size, colour, and nutritional profile yet shares core features of high-quality plant protein, complex carbohydrates, resistant starch, and a range of micronutrients and bioactive compounds [4,5,6]. As such, pulses are recognised both as nutrient-dense foods and as key components of sustainable agri-food systems because of their nitrogen-fixing capacity and relatively low environmental footprint [7]. Nutritionally, pulses are rich in complex carbohydrates, dietary fibre (including soluble fibre and resistant starch), protein, and a wide array of micronutrients such as iron, zinc, folate, magnesium, potassium, and B vitamins [6,7,8]. Pulses generally contain 2–4 times more protein than cereal grains and are especially rich in the essential amino acid lysine, which is frequently deficient in diets based mainly on cereals [9]. This makes pulses excellent candidates for complementary protein-pairing with cereals, thereby improving overall dietary protein quality and supporting the prevention of protein-energy malnutrition in low-income populations [10,11]. Pulses are also an important source of bioactive compounds, including polyphenols, saponins, phytosterols, lectins, protease inhibitors, and non-digestible carbohydrates [6,12,13]. Recent compositional analyses highlight considerable variation in protein digestibility, fibre content, and antioxidant capacity among different pulse types (e.g., black beans, lentils, chickpeas), underscoring the importance of considering specific pulse species when evaluating their health effects [14,15,16].

Pulses are increasingly recognised for their potential role in cancer risk reduction and therapy. These foods are rich in bioactive compounds such as polyphenols, flavonoids, saponins, lectins, and protease inhibitors, which have been shown to exert anti-cancer effects in a variety of preclinical models [13]. In vitro studies have shown that pulse extracts and isolated compounds can suppress cancer cell proliferation, promote apoptosis, and influence key signalling pathways involved in carcinogenesis. For example, extracts from black beans and navy beans have been found to decrease proliferation and enhance apoptosis in human colon and breast cancer cell lines, partly through the modulation of genes associated with cell cycle control and apoptosis [17,18]. Similarly, lentil-derived peptides and phenolic fractions have exhibited strong anti-proliferative activity against colon, breast, and nasopharyngeal carcinoma cells, frequently by inducing cell cycle arrest and activating caspase-dependent apoptosis [13,19]. Animal studies further support these findings. Diets supplemented with navy beans, black beans, or lentils have been shown to suppress tumour development and reduce tumour burden in chemically induced models of colon and mammary cancer in rodents [20,21]. The anti-cancer effects of pulses in vivo have been attributed to their ability to modulate oxidative stress, reduce inflammation, and favourably alter the composition of the gut microbiota [22], thus boosting the generation of short-chain fatty acids (SCFAs) such as butyrate, which exhibit anti-tumourigenic properties [23]. Collectively, these cell and animal studies provide compelling evidence that pulses harbour a diverse array of bioactive compounds capable of interfering with multiple hallmarks of cancer. These findings lay the groundwork for future translational research and support the potential of pulses as functional foods in cancer risk reduction and therapy. Existing meta-analyses of legumes and cancer aggregate legumes broadly, often combining FAO-defined pulses with soy and peanuts, and are restricted to observational incidence endpoints [24,25]. The recent World Cancer Research Fund International 2025 report addresses legumes and cancer within wider dietary and lifestyle patterns rather than examining pulses as a distinct exposure [26]. These syntheses therefore provide limited resolution on the specific contribution of pulses across the cancer continuum. Against this background, this scoping review aims to synthesise existing human evidence on the role of pulses, with a primary focus on dry beans (kidney, black, cannellini, navy and pinto), chickpeas, dry peas (split and black-eyed), and lentils, in cancer risk reduction and related mechanistic and survivorship-relevant outcomes. While these pulse types are the principal focus, studies involving other pulses will also be considered where relevant. A scoping review approach was selected rather than a systematic review with meta-analysis because the field lacks sufficient homogeneous randomised controlled trials (RCTs) to pool quantitatively, and our primary objective was to map the breadth of available evidence (quantity, characteristics, research gaps) across heterogeneous study designs (RCTs, case–control, cohorts) and cancer types rather than assess effect sizes or study quality. These pulses were selected for this scoping review because they represent the most globally recognised and commonly consumed pulses [27,28,29]. The selection serves as a starting point and is not restricted to this list, as additional pulse types may be considered where relevant. By mapping epidemiological, clinical, and biomarker-driven studies, we seek to clarify mechanisms (e.g., modulation of the gut microbiota, anti-inflammatory effects) and identify research gaps, thereby informing future interventions that leverage pulses as functional foods in oncology.

## 2. Materials and Methods

The literature search was conducted using Cochrane Central Register of Controlled Trials (CENTRAL), Scopus, and PubMed for original articles published from 2014 to December 2025 (last search: 31 December 2025). Studies published after 31 December 2025 were not eligible for inclusion in the scoping review but, where relevant, more recent 2026 studies were cited as external contextual evidence without being part of the formal study set. In addition, backward and forward citation tracking of all eligible articles was performed to identify further studies not captured in the database searches. This scoping review adhered to the Preferred Reporting Items for Systematic reviews and Meta-Analyses extension for Scoping Reviews (PRISMA-ScR) guidelines [30] and no pre-published protocol was registered (Table S1). The search strategy was guided by the Population, Concept, and Context (PCC) framework developed by the Joanna Briggs Institute (JBI) for scoping reviews [31].

Population: Human participants, including cancer patients, survivors, at-risk individuals (e.g., polyp patients, high-risk cohorts), and general populations in epidemiological studies examining cancer risk.

Concept: Role of pulses (dry beans (navy, kidney, black, pinto), lentils, and dry peas (split, black-eyed), chickpeas) and their effects on cancer risk reduction and related mechanistic and survivorship-relevant outcomes (for example biomarkers, microbiome modulation, metabolites, or symptom-related measures), where available.

Context: Human clinical studies (RCTs, cohort, case–control, controlled feeding, pilot interventions) published 2014–2025 in English, full-text, peer-reviewed journals focusing on cancer-specific outcomes.

The search strategy employed broad and synonym-inclusive terms for pulses (e.g., “beans,” “lentils,” “peas,” and related variants) to maximise sensitivity and capture the diverse pulse types reported in the literature. While key pulse categories (dry beans (navy, kidney, black, pinto), lentils, and dry peas (split, black-eyed), chickpeas) are highlighted for conceptual clarity and data synthesis, the search strategy was not restricted to these specific types and did not intentionally exclude other pulses (e.g., common beans, mung beans, adzuki beans). Relevant keywords were used to identify studies examining the role of pulses in cancer-specific outcomes among human populations, and the reference lists (backward citation) and cited articles (forward citation) of included studies were screened to capture additional eligible records. Only full-text articles published in English were included. Studies were excluded if they: (i) were conducted exclusively in vivo or in vitro; (ii) did not include pulses as a clearly defined primary exposure, intervention, or prespecified component of a dietary pattern; (iii) evaluated broader dietary patterns without the possibility of attributing effects to pulses; (iv) focused solely on the compositional analysis or bioactive constituents of pulses without reporting any cancer-specific clinical outcomes or cancer-related biomarkers; (v) were reviews, meta-analyses, protocols, conference abstracts, letters, commentaries, or other non-peer-reviewed sources; (vi) did not report cancer-specific clinical outcomes or cancer-related biomarkers; (vii) were multi-component dietary or pharmacological interventions in which pulses were administered alongside other co-interventions and the effects of pulses could not be isolated. Observational studies with legume exposures composed predominantly of FAO-defined pulses but containing unspecified preparation for some items (for example peas) were classified as pulse-dominant and retained, with their findings interpreted as predominantly, rather than exclusively, pertaining to pulses. These restrictions on study type, language, and publication period were applied to ensure methodological rigour, feasibility of screening, and a focus on contemporary evidence relevant to cancer risk reduction and to mechanistic and survivorship-relevant outcomes. The full search strategy is listed in Table 1.

**Figure 1:**
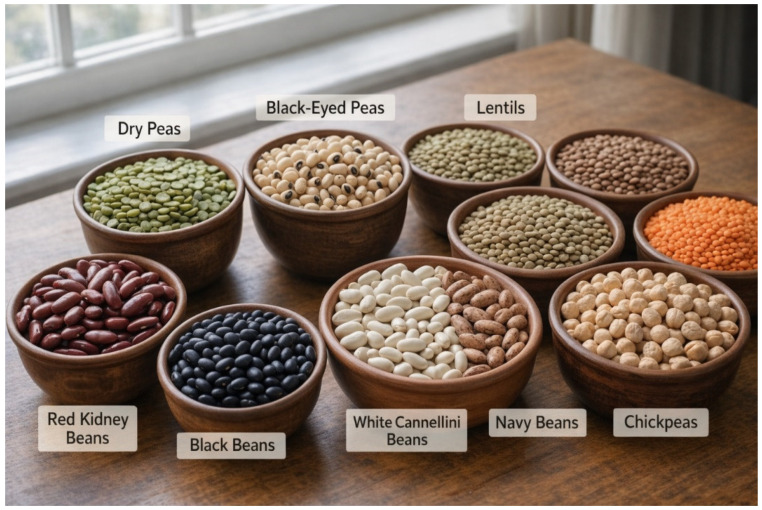
Pulses selected for this scoping review. The authors declare the use of generative AI (Gemini 3) to generate this image.

The pulses selected for categorization and presentation in this study are shown in Figure 1. These reflect the primary pulse types identified in the included studies and are not intended to represent an exhaustive or restrictive list of all pulses considered in the search strategy.

Four independent reviewers (M.N.M.N, N.I., T.B.Y. and V.R.B.) screened titles and abstracts (exported from CENTRAL, Scopus, and PubMed, and backward, forward citation searches via EndNote Version X9 with duplicates removed) against predefined PCC-based criteria, with discrepancies resolved by group consultation and final decisions by M.N.M.N. Full-text articles were assessed similarly. Data were charted in duplicate by three reviewers (N.I., T.B.Y. and V.R.B.) using a piloted Excel form capturing study objectives, design, population characteristics, pulse exposure and preparation, cancer-related outcomes, key findings, and authors’ conclusions. Each study’s extraction was independently performed by two reviewers and then cross-checked, with disagreements resolved by discussion and final verification by M.N.M.N. Charted data were then collated and synthesised narratively, with studies grouped by cancer site, study design and population, pulse exposure and preparation, and cancer-related outcomes (risk, progression/biomarkers, survivorship) to identify patterns, consistencies, and evidence gaps. Consistent with the scoping review methodology, no formal critical appraisal was undertaken, as the objective was to map the breadth and characteristics of the evidence rather than assess individual study quality.

## 3. Results

**Figure 2:**
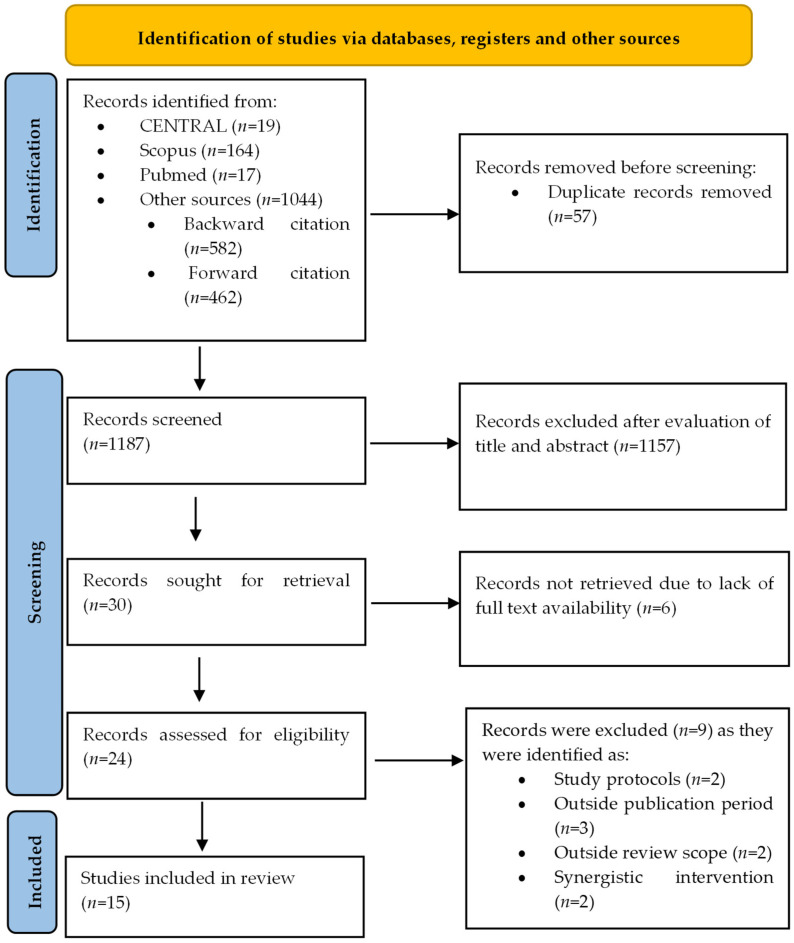
PRISMA flow diagram.

The search identified 1244 records, including 200 from electronic databases (CENTRAL, Scopus, PubMed) and 1044 from other sources (backward citation tracking, *n* = 582; forward citation tracking, *n* = 462). After removal of 57 duplicate records, 1187 unique records were screened at the title and abstract level, of which 1157 were excluded. The remaining 30 records were sought for retrieval; six could not be obtained due to lack of full-text availability, leaving 24 records for full-text assessment. At this stage, 9 records were excluded (study protocol, *n* = 2; outside publication period, *n* = 3; outside review scope, *n* = 2; synergistic intervention, *n* = 2), resulting in 15 studies being included in the final synthesis of this scoping review. The study selection process is illustrated in the PRISMA flow diagram, showing the number of records identified, screened, assessed for eligibility, and included (Figure 2).

**Figure 3:**
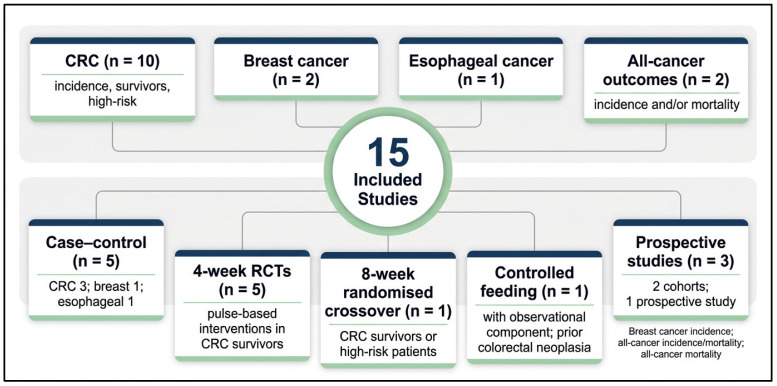
Overview of included studies.

The 15 included studies predominantly investigated colorectal cancer (CRC) (10 studies), with others examining breast cancer (2 studies), oesophageal cancer (1 study), and cancer mortality for all cancer sites combined (2 studies). The evidence base comprised five case–control studies (three on CRC, one on breast cancer, and one on oesophageal cancer), five 4-week RCTs, and one 8-week randomised crossover trial of pulse-based interventions in CRC survivors or high-risk patients. In addition, one controlled feeding study with an observational component was conducted in men with prior colorectal neoplasia, two prospective cohort studies examined breast cancer incidence and all-cancer incidence and mortality, and one prospective study evaluated pulse intake in relation to all-cancer mortality, giving a total of 15 studies. Figure 3 provides an overview of the included studies in this review.

Pulses investigated across these studies included navy beans in five interventions, mixed dry beans (pinto, navy, kidney, lima and black, including baked beans) in one controlled feeding study, lentils in three studies, peas in three studies, chickpeas in two studies, and broader “pulses” or mixed bean–lentil–chickpea categories in three observational studies. In the intervention work, pulses were typically provided as cooked whole beans that were pressure-cooked and canned, or as cooked beans that were dried, ground and incorporated into meals and snacks, whereas the observational studies assessed usual intake of cooked or canned pulses without detailed information on preparation methods. Several observational studies used the umbrella term “legumes”, but inspection of their dietary instruments indicated that reported intake consisted exclusively or predominantly of pulses (dried beans, lentils, chickpeas, dry peas), with soybeans, peanuts and other non-pulse legumes either excluded or assessed separately; in some of these legume exposures, items such as peas were included without specification of fresh versus dried form, and were therefore treated as pulse-dominant rather than exclusively pulses. For clarity and consistency, we use the term “pulses” or specific pulse types (e.g., dried beans, lentils, chickpeas, dry peas) throughout this manuscript when referring to the exposure assessed, while retaining original study terminology when directly quoting findings. A more detailed breakdown of study designs, pulse exposures and preparation methods, cancer sites, outcomes and main findings is presented in Table 2, which also reports the reference, year and country for each study.

## 4. Discussion

### 4.1. Cancer Outcomes

#### 4.1.1. Colorectal Cancer Risk

Globally, CRC ranks within the top three most common malignancies and is the second major cause of cancer mortality, accounting for roughly 1.9 million incident cases and more than 900,000 deaths in 2020 [47]. Its global burden is closely linked to dietary patterns, and low intake of fibre-rich plant foods, including pulses, is now recognised as a major modifiable contributor to CRC incidence and mortality [48], with deaths attributable to low-fibre diets increasing by over one-third worldwide between 1990 and 2021 [49]. Within this context, CRC is the cancer site most frequently examined in relation to pulse intake in the included studies, spanning both primary risk reduction in the general population and supportive interventions among CRC survivors and high-risk individuals. The three included observational studies provide the only CRC risk data including predominantly FAO-defined pulses, albeit with some items of unspecified form. Two Jordanian case–control studies [32,33] in hospital settings, involving 220 CRC cases with 281 controls and 167 CRC cases with 240 controls, evaluated habitual intake of lentils and peas. In one, higher intake of lentils and peas suggested a protective trend against CRC, but associations were not statistically significant. As the form of peas was unspecified, this exposure is treated as pulse-dominant rather than exclusively pulses [32]. In the other, weekly lentil intake showed no significant association with CRC risk [33]. By contrast, a large pooled analysis from Italy and Switzerland that a combined total of 10,482 cases across 10 cancer sites reported a clear inverse association for CRC: compared with non-consumers, individuals consuming at least one portion of pulses per week had an odds ratio (OR) of 0.79, and those consuming two or more portions per week had an odds ratio of 0.68, with an estimated 13% reduction in CRC risk for each additional weekly portion [34]. These findings indicate that while some case–control datasets show only suggestive or null relationships, others demonstrate a robust dose–response pattern linking higher pulse intake with lower CRC risk. Beyond the included studies, several external case–control investigations examine legume-rich dietary patterns and CRC, though these use broad legume categories may or may not include pulses as the primary component, and pulse-specific conclusions cannot be drawn from them.

A Portuguese case–control study of 151 rectal and 102 colon cancer cases identified a “healthy” dietary pattern characterised by higher intakes of legumes, vegetables, fruits, wholegrains and dairy, which was associated with substantially lower odds of CRC compared with both a low-fibre, low-milk pattern and a Western pattern [50]. Similar findings were reported from Iran, where principal component analyses of dietary patterns showed that a “healthy” pattern rich in fruits, vegetables, legumes and wholegrains was associated with markedly reduced CRC odds, whereas more traditional or Western patterns higher in refined carbohydrates and animal products were associated with increased risk [51,52]. These external data therefore reinforce the notion that legumes, considered as part of broader fibre- and plant-rich dietary patterns, tend to cluster with lower CRC incidence. However, the legume–CRC association is complicated by heterogeneous findings across external meta-analyses and individual cohort studies. A recent systematic review and meta-analysis of 29 observational studies on legumes and CRC [24] reported that, although pooled estimates for the highest versus lowest legume intake categories were below unity (Relative Risk (RR): 0.90, 95% Confidence Interval (CI): 0.83–0.98) and dose–response analyses suggested approximately a 21% lower CRC risk per 100 g/day increment, the overall evidence was graded as weak due to substantial heterogeneity, regional variation and attenuation of effect estimates when analyses were stratified by rigorous adjustment for body mass index (BMI), smoking, alcohol, red and processed meat, and other dietary factors. Importantly, most of these studies measured legumes as a broad exposure category and often combined pulses with soy products and peanuts, making it difficult to attribute any observed associations specifically to pulses [24,25]. Moreover, several high-quality cohort and case–control studies focusing on overall dietary patterns rather than individual foods show that CRC risk is more consistently elevated in Western, low-fibre patterns and reduced in “healthy” patterns than it is associated with legume intake per se, suggesting that legumes may act primarily as markers of more favourable dietary profiles rather than as independent protective factors [50,51].

Taken together, the three included observational studies indicate that higher pulse or pulse-dominant legume intake is compatible with lower CRC risk, but the evidence is mixed: Jordanian case–control data show, at best, non-significant protective trends [32,33], while the Italian–Swiss network demonstrates a clear dose–response relationship in favour of higher pulse intake [34]. External epidemiological data from other case–control and cohort studies broadly support a modest inverse association between legume-rich, high-fibre dietary patterns and CRC, yet they also reveal that these associations are sensitive to the way diet is measured, to confounder adjustment, and to regional dietary contexts [24,50,51]. Critically, because most datasets use aggregated “legume” categories that encompass pulses alongside soy and other legumes, and because legumes frequently co-occur with other protective foods in healthy dietary patterns, it is not possible to ascribe the observed reduction in CRC risk specifically to pulses [24,25]. This distinction, combined with the heterogeneity in study designs and populations, argues for a cautious interpretation: legumes as a broad group appear to contribute to dietary patterns associated with lower CRC incidence, but the strength and independence of any protective effect attributable to pulses alone remains uncertain and should not be overstated [26].

#### 4.1.2. Breast Cancer Risk

Breast cancer accounted for approximately two million new diagnoses globally in 2020, and modifiable lifestyle factors, such as diet, are now recognised as meaningful contributors to risk modulation. The present scoping review identified two observational studies providing contrasting yet complementary perspectives on the pulse–breast cancer relationship. Although both studies described their exposures as ‘legumes’, the component foods were predominantly FAO-defined pulses (lentils, chickpeas, dry beans). As the form of some items (e.g., peas, lima beans) was not specified, some of the intake may have involved non-dried preparations, so we interpret these as signals for a pulse-dominant legume intake rather than for pulses alone. In a population-based case–control study of 350 Iranian breast cancer cases and 700 controls, women in the highest tertile of legume intake (lentils, chickpeas, peas, pinto and red beans) had 46% lower odds of breast cancer than those in the lowest tertile, with particularly pronounced inverse associations in postmenopausal (OR: 0.51) and normal-weight women (OR: 0.49) and no meaningful association in premenopausal or overweight, obese women [42]. By contrast, in the Nurses’ Health Study II, a 20-year prospective cohort of 88,803 predominantly premenopausal women, legume intake (beans, lentils, peas and lima beans), analysed as an independent exposure, was not significantly associated with incident breast cancer, whereas substituting one daily serving of this legume group for one serving of total red meat was associated with a 15% overall reduction in breast cancer risk and a 19% reduction among premenopausal women [43]. It is important to note, however, that the legumes examined in both studies, which were predominantly pulse species, contain negligible or far lower isoflavone concentrations than soybeans [53,54]. The phytoestrogenic mechanisms commonly attributed to soy therefore cannot be straightforwardly applied to interpret these findings, and the biological basis of any protective association with pulse-dominant legume exposures specifically remains less clearly defined [26,55].

The pattern of findings across the two included studies is most plausibly framed through two pulse-relevant mechanisms: glycaemic regulation and dietary displacement. Pulses have among the lowest glycaemic index values of any starchy food, and their high soluble fibre and resistant starch content attenuates postprandial insulin and glucose responses, thereby reducing circulating insulin and insulin-like growth factor-1 (IGF-1)—hormonal axes that promote mammary cell proliferation and are associated with breast cancer risk, particularly in overweight and obese women [56,57,58]. This may partly explain why the protective association with legumes in the work of Sharif et al. [42] was confined to normal-weight rather than overweight or obese women: in the latter group, peripheral oestrogen aromatisation in adipose tissue and associated insulin resistance may represent a dominant risk pathway that attenuates the modest glycaemic benefit of legume consumption. The dietary displacement mechanism is potentially illustrated by Farvid et al. [43], whose findings are consistent with a broader body of evidence showing that each additional daily serving of red meat is associated with a 13–22% higher breast cancer risk depending on the life stage of exposure. Substituting legumes for red meat may therefore simultaneously remove a pro-carcinogenic food and introduce a nutrient-dense, low-glycaemic alternative [43,59]. At the dietary pattern level, the 2025 US Dietary Guidelines Advisory Committee graded the evidence that patterns high in legumes, vegetables, fruits and whole grains and low in red and processed meat are associated with lower postmenopausal breast cancer risk as moderate-certainty, with a less consistent but directionally similar effect for premenopausal disease [60].

Several caveats constrain the strength of inference from the available data. The protein substitution analysis by Farvid et al. [43] cannot attribute the protective association to pulses or legumes per se, since the benefit may arise from the elimination of red meat rather than from any intrinsic property of this legume group; the absence of a significant independent pulse–breast cancer association in that same cohort reinforces this interpretation. The subgroup-dependent pattern in the work of Sharif et al. [42], with significant associations observed only in postmenopausal and normal-weight women, suggests that the relationship is modified by hormonal and metabolic context rather than reflecting a universal chemoprotective effect of pulses or legumes. The glycaemic hypothesis, while biologically plausible, has yielded inconsistent results in observational data: several large prospective studies have found no significant independent association between dietary fibre intake and breast cancer risk, and the association between glycaemic index or glycaemic load and breast cancer was statistically significant in some cohorts but null in others after full adjustment [61,62,63]. These inconsistencies suggest that glycaemic modulation is, at most, one contributing pathway rather than a dominant mechanism, and that its relevance may depend on body weight and menopausal status.

The bioactive constituents of the pulses that are most plausible for breast cancer risk modulation, including fibre, polyphenols, phytosterols and resistant starch, were not tested in human breast cancer populations at dietary doses in any included study, and the two epidemiological studies included here therefore provide signals without a corresponding mechanistic evidence base [25,26]. Taken together, the available data suggest a plausible but incompletely characterised relationship between pulse-dominant legume exposures and breast cancer risk, best explained through improved glycaemic regulation, enhanced dietary quality and displacement of carcinogenic foods rather than through legume or pulse-specific pharmacological mechanisms. Given that both studies relied on pulse-dominant legume exposures in which the form of some items was not specified, and that one protective signal was confined to specific subgroups while the other arose from a substitution model rather than an independent effect, these findings should be interpreted cautiously. They are best regarded as preliminary signals for a pulse-dominant dietary pattern rather than as evidence of a direct causal role of pulses in breast cancer risk reduction.

#### 4.1.3. Oesophageal Cancer Risk, and Other Cancer Outcomes

Oesophageal cancer has one of the poorest prognoses among solid malignancies, with five-year survival below 20% in many settings, making dietary risk reduction strategies particularly salient. Only one included study examined oesophageal cancer in relation to pulse intake. In a large hospital-based case–control study from South Africa including 670 oesophageal cancer cases and 1188 controls, dry bean consumption was not significantly associated with oesophageal cancer risk in either men or women [44]. Null findings for dry beans may reflect non-differential exposure misclassification, as bean intake was classified solely by frequency without accounting for portion size or preparation, a limitation likely to bias risk estimates toward the null. It should be noted that Sewram et al. [44] also assessed green legumes (peas and green beans), for which a protective association was observed in women. However, green legumes harvested and consumed fresh fall outside the FAO definition of pulses, and this signal therefore cannot be attributed to pulses specifically.

Broader evidence on plant-rich diets provides context for the null pulse finding, but does not alter its interpretation. A meta-analysis of 32 observational studies encompassing 10,037 oesophageal squamous cell carcinoma (ESCC) cases reported pooled summary RR of 0.56 (95% CI: 0.45–0.69) for high versus low vegetable intake and 0.53 (95% CI: 0.44–0.64) for fruit intake, with no indication of publication bias [64]. Case–control nutrient analyses have similarly shown that patients with oesophageal or gastric cancer tend to have lower intakes of fibre, beta-carotene, folate, vitamin C, vitamin B6, while higher intakes of animal protein and cholesterol are over-represented in cases [65]. In high-incidence Southern African regions, ESCC risk has also been linked to heavy reliance on maize meal as a staple. The hydrolysis of esterified linoleic acid in stored maize produces free linoleic acid, which can increase gastric prostaglandin E2 synthesis and promote low-acid duodenogastro–oesophageal reflux, thereby exposing oesophageal mucosa to carcinogenic insults [66]. In such contexts, frequent green legume intake may operate less through legume-specific pharmacological effects and more as a marker of dietary diversity that supplies fibre and micronutrients while partially displacing maize-based staples.

The existing evidence is insufficient to establish pulses as an independent protective factor for oesophageal cancer. The 2018 World Cancer Research Fund/American Institute for Cancer Research Continuous Update Project concluded that the data specifically implicating legumes, considered separately from fruits and vegetables, in ESCC risk reduction are too limited and inconsistent to underpin focused dietary recommendations [67]. Furthermore, evidence from African ESCC settings highlights multiple co-occurring risk factors, including alcohol, very hot beverage consumption, poor oral health and biomass fuel exposure, making it unlikely that any single food group explains a large fraction of risk [68]. A 2014 meta-analysis of dietary patterns reported that “prudent” patterns were inversely associated with ESCC, but between-study heterogeneity was substantial, again suggesting that no single dietary component can be reliably isolated as the operative protective element [69]. On the basis of the single included study and its null pulse-specific findings, this review cannot support pulse-based dietary recommendations for oesophageal cancer risk reduction. Longer-term prospective studies with detailed pulse-specific dietary assessments are needed to address this evidence gap.

Prospective cohort studies offer a broader perspective on pulses and cancer-related survival across sites. In a Spanish population-based cohort of 66,933 adults drawn from three national surveys, categorising pulse intake as <1, 1–2, or >3 times per week was not associated with cancer mortality after adjustment for health and lifestyle factors [45]. In contrast, a cohort study of 41,243 Chinese adults initially free of cancer and cardiovascular disease (CVD) reported inverse associations between legume intake (beans, lentils, chickpeas, peas and black-eyed peas—a pulse-dominant legume group) and both cancer incidence and mortality in minimally adjusted models, with approximately 29% lower cancer mortality among those consuming one to two servings of this legume group per week. Because the form of some items (e.g., peas) was not specified, some intake may have involved non-dried preparations, and these findings are therefore interpreted as signals for pulse-dominant legume intake rather than for pulses alone [46]. However, these associations were substantially attenuated and lost statistical significance after more extensive adjustment for socioeconomic and lifestyle covariates. The attenuation of pulse–cancer mortality associations in fully adjusted models does not necessarily exclude a true biological effect but underscores the challenge of isolating pulses from overall diet quality. Beyond the two included studies, external evidence on broader legume intake and cancer mortality provides useful context, though pulse-specific conclusions cannot be drawn from it. A systematic review and dose–response meta-analysis of 32 prospective cohort studies, encompassing 1,141,793 participants and 93,373 all-cause deaths, demonstrated that higher legume intake was associated with a 6% reduction in the risk of all-cause mortality per 50 g/day increment (Hazard Ratio (HR): 0.94, 95% CI: 0.91–0.98) and a significant reduction in stroke mortality, whereas the pooled estimate for cancer mortality was directionally protective but narrowly missed significance (HR: 0.85, 95% CI: 0.72–1.01) [70]. Consistent with this evidence, the Nordic Nutrition Recommendations 2023 concluded that increased legume intake is associated with decreased risk of mortality and several site-specific cancers, including gastric, colorectal, breast and lung cancer, though these recommendations are based on broad legume categories and cannot be attributed to pulses specifically [71]. Large cohort analyses of plant-based and Mediterranean dietary patterns, in which legumes broadly are a core component, similarly demonstrate lower overall cancer risk and reduced multimorbidity involving cancer and cardiometabolic conditions, with metabolomics data identifying candidate mediators of these associations [72,73]. These external data are cited as contextual background and do not constitute pulse-specific evidence.

Nonetheless, several structural features of the available cohorts constrain causal interpretation. In the work of Martínez-Castañeiras et al. [45], pulse intake was captured using only three broad frequency categories without information on pulse type or preparation, and was based on single cross-sectional dietary assessments, which likely introduced nondifferential misclassification and bias toward the null. The analysis by Liu et al. [46] vividly illustrates the sensitivity of pulse-dominant legume–cancer mortality associations to covariate adjustment. The disappearance of statistical significance after full adjustment suggests that higher-pulse consumers differ systematically from lower consumers in unmeasured confounders, indicating classic healthy-user bias. Consistent with this, the meta-analytic evidence shows robust associations for all-cause and stroke mortality but only borderline protective effects for cancer mortality specifically [70], implying that any cancer-specific survival benefit is probably modest and embedded within generally healthier dietary and lifestyle patterns and that it cannot be attributed with certainty to pulses as distinct from pulse-dominant legume intake overall. Cancer mortality endpoints are also influenced by tumour biology, stage at diagnosis, treatment access, comorbidities and functional status, factors that are often imperfectly measured or absent in nutritional cohorts. In this context, the cohort findings are best interpreted as mapping associations for diets in which pulses and other legumes feature prominently, rather than as establishing pulses as independent determinants of cancer outcomes. Taken together, these observations are most consistent with a scenario in which pulses and pulse-dominant legume intake contribute at most modestly to better cancer outcomes as one component of overall diet quality, rather than acting as strong, independent determinants of cancer incidence or mortality.

### 4.2. Metabolite and Microbiome Responses to Pulse Intake

Mechanistic and feasibility work in CRC survivors and at-risk populations provides the most direct biological evidence for how pulses, particularly navy beans, may shape cancer-relevant physiological processes [35,36,37,38,39]. Pulses provide not only protein and low-glycaemic carbohydrates but also substantial amounts of non-digestible fibre and resistant starch, together with polyphenols and other phytochemicals [8,74,75] (flavonoids, phenolic acids, phytosterols, saponins, lectins and phytic acid), which have been implicated in anti-inflammatory, antioxidant and antiproliferative effects in experimental models [74,76,77]. However, the intervention studies included in this review did not systematically profile these bioactive fractions in the test foods, so their contribution to the observed microbiome, metabolite and gene expression changes must be inferred from the external compositional and preclinical literature rather than from direct measurements.

Across multiple randomised four-week trials in overweight or obese CRC survivors, daily provision of 35 g cooked navy bean powder was feasible to incorporate into study meals and snacks, produced meaningful increases in total dietary fibre and selected micronutrient intakes, and in some instances altered gut microbial composition and stool, plasma and urine metabolite profiles [35,36,37,38,39,40,41]. It should be noted that five of these trials [35,36,37,40,41] were conducted by the same research group using near-identical protocols and populations, which limits the independence of replication. In addition to these navy bean powder trials, a controlled feeding study in which participants consumed 250 g/day of cooked mixed dry beans [38] and the eight-week Beans to Enrich the Gut microbiome vs. Obesity’s Negative Effects (BE GONE) crossover trial, in which adults with prior CRC or advanced colorectal polyps added two servings of canned navy beans to their habitual diet [39], extended these findings to include changes in circulating metabolites, gut microbiome diversity and selected immune and inflammatory markers, with most effects regressing toward baseline once the bean intervention was withdrawn.

Collectively, this body of evidence, spanning case–control biomarker work, controlled feeding studies and crossover survivor trials, constitutes the most mechanistically detailed clinical foundation currently available for the role of pulses in CRC care. Mechanistic pathways discussed in the following subsections are therefore inferred from the external preclinical literature to contextualise the human trial findings and do not constitute evidence from the included studies, and human trials directly testing these mechanisms at dietary pulse doses are currently absent. In the following subsections, we therefore examine in turn: (i) microbiome diversity and taxa shifts (Section 4.2.1), (ii) plasma, urine and stool metabolite signatures and pathways (Section 4.2.2), (iii) short-chain fatty acid dynamics and colonocyte biology (Section 4.2.3), and (iv) emerging evidence for pulse-induced changes in gene expression and epigenetic regulation (Section 4.2.4), highlighting both convergent patterns and key gaps that limit causal inference.

#### 4.2.1. Microbiome Diversity and Taxa

Pulse consumption has a demonstrable capacity to reshape gut microbial communities in CRC survivors and high-risk individuals, though the extent and functional significance of this remodelling depend markedly on dose, delivery form and intervention duration. In a four-week randomised trial in which overweight or obese CRC survivors consumed 35 g/day of cooked navy bean powder, stool bacterial richness increased by day 28, whereas overall diversity indices, community structure, phylum-level composition, predicted functional gene content and stool short-chain fatty acid concentrations remained statistically unchanged [35]. A different pattern emerged from the BE GONE randomised crossover trial [39], in which adults with a history of CRC or advanced colorectal neoplasia added approximately two servings of canned navy beans per day to their habitual diet for 8 weeks: participants experienced significant increases in microbial alpha diversity and shifts in community composition, including expansion of *Faecalibacterium*, *Eubacterium* and *Bifidobacterium* and contraction of several potentially pro-inflammatory or opportunistic taxa such as *Roseburia*, *Streptococcus*, *Collinsella*, *Escherichia*, *Ruminococcus torques*, *Fournierella* and *Oscillibacter*. These microbiome changes were largely reversible, with alpha diversity and taxonomic profiles regressing toward baseline after participants returned to a bean-free diet during the washout phase, indicating that sustained intake is required to maintain the prebiotic effect. The genus-level shifts observed in the BE GONE trial are consistent with broader microbiome patterns reported in CRC and preclinical models. *Faecalibacterium prausnitzii* and *Bifidobacterium* are typically reduced in patients with CRC compared with healthy controls and are frequently linked to butyrate production, anti-inflammatory effects and improved epithelial barrier function [78,79,80,81]. Complementing these, a retrospective cohort study in 80 patients with advanced CRC reported that 12 weeks of combined dietary fibre and multi-strain probiotic supplementation significantly increased Shannon Diversity Index and the relative abundance of *Bifidobacterium* and *Lactobacillus*, and was associated with higher three-year overall survival (83.5% vs. 67.4% in controls), suggesting that microbiome diversification can translate into clinically meaningful survival benefits [82].

Food structure may also influence these microbiome responses. In vitro digestion–fermentation studies suggest that intact bean cotyledon cell walls and protein matrices slow starch digestion in the upper gut and increase the delivery of fermentable substrate to the colon, whereas disruption of cotyledon integrity accelerates starch hydrolysis more proximally [83]. The discrepancy between the powder and whole-bean trials therefore warrants cautious interpretation. The absence of community-level diversity and stool SCFA changes in the navy bean powder trial [35], despite the increased bacterial richness and fibre intake, underscores that richness alone is an imperfect proxy for functional microbiome remodelling and that food structure could partially explain these divergent findings. The reversibility of the BE GONE trial [39] microbiome changes after bean withdrawal further highlights an unresolved translational challenge, as no available trials have tested adherence to bean intakes of this magnitude beyond 8 weeks and all were conducted in predominantly overweight or obese North American CRC survivors with low-fibre Western dietary patterns, limiting generalisability to other populations.

#### 4.2.2. Metabolite Signatures and Pathways

Across multiple trials, navy bean interventions produced consistent and reproducible alterations in the stool, plasma, urine and serum metabolomes of CRC survivors, with characteristic signatures reflecting both direct bean-derived metabolites and downstream host–microbiome interactions. In an 18-participant randomised trial, 35 g/day of navy bean powder altered 31 stool metabolites compared with the baseline and 26 versus macronutrient-matched controls; among 237 potential bean-derived metabolites, pathway analyses revealed a greater than fivefold elevation in stool ophthalmate, changes in lysine, histidine and branched-chain amino acid degradation pathways, shifts in medium-chain fatty acid, glycerolipid and inositol metabolism, and concurrent rises in antitumour phytochemicals, including piperidine, enterolactone and nobiletin [36]. Complementary plasma and urine metabolomics in a separate four-week trial in 20 CRC survivors showed that navy bean powder elevated plasma 2,3-dihydroxy-2-methylbutyrate, pipecolate and S-methylcysteine and raised urinary N2,N5-diacetylornithine and 4-hydroxyhippurate [37]. In a controlled feeding crossover in 46 men consuming 250 g/day of cooked dry beans, serum pipecolic acid and S-methylcysteine were the most sensitive biomarkers of bean intake, with 80 serum metabolites significantly altered after 4 weeks and these 2 analytes maintaining strong longitudinal correlations with bean consumption across multiple years [38]. The BE GONE trial corroborated these signatures, observing increases in circulating pipecolic acid, S-adenosyl-l-methionine (SAM) and trigonelline during the bean phase that reversed within 4 weeks of withdrawal [39]. A comprehensive plasma, urine and stool metabolomics analysis further confirmed that S-methylcysteine, pipecolate, 4-methoxyphenol sulphate and 2,3-dihydroxy-2-methylbutyrate are consistently elevated across both bean and rice bran dietary interventions in CRC-relevant populations, underscoring the reproducibility of these signatures [84]. Notably, although S-methylcysteine, pipecolate and trigonelline are recognised signatures of dry bean intake likely reflecting underlying polyphenolic and sulphur-containing bioactives, the specific compound profiles of navy bean test foods were not quantified in any included trial, so the links between individual metabolites and discrete bioactive fractions remain hypothesis-generating.

Stool ophthalmate, a glutathione analogue elevated more than fivefold in the Baxter et al. trial [36], has been proposed as a marker of altered glutathione flux and redox homeostasis [85,86,87]. Pipecolic acid and S-methylcysteine have been validated as non-invasive biomarkers of dry bean intake in both human and murine models, and their consistent appearance across navy bean interventions identifies them as candidate adherence markers for future longer-term trials. Independently, pipecolic acid has been implicated as one of several serum metabolites with potential utility for monitoring therapeutic response and disease relapse in ESCC, but such applications remain preliminary [88].

#### 4.2.3. Short-Chain Fatty Acids (SCFAs) and Colonocyte Biology

The SCFA data from the navy bean trials present a more complex picture than a straightforward fibre-to-butyrate model. In the work of Sheflin et al. [35], a four-week navy bean powder trial in CRC survivors, substantial increases in dietary fibre intake and bacterial richness were not accompanied by measurable changes in stool SCFA concentrations, diverging from much of the prebiotic literature and raising questions about whether standard stool assays reliably capture colonic SCFA dynamics at this dose and duration. Butyrate’s role as an energy substrate for normal colonocytes and as an HDAC inhibitor in neoplastic cells is among the best-characterised examples of diet–microbiome–epithelium crosstalk in CRC [89,90,91,92,93,94,95,96,97]. Within the included human studies, the null stool SCFA findings in Sheflin et al. [35] and Baxter et al. [36] are most plausibly explained by a combination of sampling limitations and context-specific responses: SCFAs produced in the proximal colon are extensively consumed at the mucosal surface and may not accumulate in distal stool, and faecal butyrate responses to resistant-starch-rich diets show substantial inter-individual variability linked to baseline microbiome composition [98,99,100,101,102,103]. Food structure and microbial community shifts may further contribute to the mixed SCFA findings but remain incompletely characterised in humans. Milling navy beans into a fine powder disrupts cotyledon cell architecture and increases starch digestibility in the small intestine, potentially reducing resistant starch delivery to the colon compared with whole-bean formats [104,105,106,107,108]. In the BE GONE trial [39], a reduction in *Roseburia*, a recognised butyrate-producing genus [109,110], alongside increases in other taxa suggests that navy bean intake may reshuffle microbial communities in ways that obscure net SCFA changes at the stool level. Taken together, the available data suggest that pulses have the capacity to influence SCFA-related pathways and more targeted studies with mucosal or portal measurements will be needed to clarify tissue-level SCFA effects.

#### 4.2.4. Gene Expression and Epigenetic Regulation

At the molecular level, enrichment of the diet with pulses has been shown in controlled feeding and crossover studies to alter gene expression in colonic epithelial cells and may indirectly modulate epigenetic regulatory processes, although these endpoints were measured less frequently than metabolomic outcomes across the included studies. In the dry bean-enriched controlled feeding study that complemented the biomarker work of Perera et al. [38], the transcriptional profiling of exfoliated colonocytes from participants consuming 250 g per day of cooked mixed dry beans over 4 weeks revealed modulation of genes governing cell-cycle progression, oxidative stress response, and xenobiotic detoxification, collectively consistent with a shift toward enhanced epithelial differentiation and reduced oncogenic signalling [38]. The observation in the BE GONE trial [39] of elevated circulating SAM during the bean consumption phase introduces a plausible epigenetic dimension to these findings. SAM, the principal methyl donor for DNA, RNA, and histone methylation in mammalian cells, is rate-limiting for tumour-suppressor gene methylation fidelity and the restoration of hypomethylated CRC-associated gene promoters.

Global DNA hypomethylation and aberrant CpG island hypermethylation are early and consistent features of CRC, and SAM, as a universal methyl donor, has been shown in CRC cell lines and other tumour models to modulate proliferation, cell-cycle progression, DNA repair, and metastasis-related pathways via transcriptional and gene-specific methylation mechanisms [111,112,113]. Beyond SAM, non-digestible fractions of common beans have been reported in animal and cell culture models to influence colonic gene expression patterns consistent with cell cycle arrest, apoptosis induction, and modulation of antioxidant response pathways, suggesting that multiple bean-derived components may contribute to epigenetic and transcriptomic effects observed in human trials [114,115,116,117].

### 4.3. Diet Quality and Micronutrient Enrichment

Navy bean-based interventions in CRC survivors demonstrate that pulse inclusion can substantially improve overall diet quality at realistic intake levels. In four-week randomised trials where survivors consumed 35 g/day of cooked navy bean powder incorporated into one study meal and one snack, total dietary fibre intake increased between week two and week four among CRC survivors, and navy bean-containing meals provided higher intakes of iron, magnesium, zinc, folate, vitamin B6, calcium, and potassium compared with macronutrient-matched control foods [40,41]. In non-cancer participants from the feasibility trial [41], total energy and carbohydrate intakes were significantly lower at week four in the navy bean group compared with the baseline; while this pattern is consistent with a satiety effect, it should be interpreted with caution given the very small non-cancer subsample (*n* = 7) and the absence of a parallel response in CRC survivors, who increased carbohydrate intake over time without a corresponding increase in total energy intake. In the feasibility trial including CRC survivors and non-cancer adults, GI discomfort was reported as minimal and similar between navy bean and control meals, and the Vege Full™ navy bean powder was described as a practical, nutrient-rich way to increase bean intake [41], although no other included intervention systematically evaluated GI symptom burden.

These intervention findings align closely with large-scale observational and modelling studies. Two 2024 studies using National Health and Nutrition Examination Survey (NHANES) data from 2001 to 2018 support the nutrient density benefits of bean consumption in US dietary patterns. A modelling study found that adding one or two daily bean servings to the typical US dietary pattern was associated with significantly higher intakes of dietary fibre, potassium, magnesium, iron, folate, and choline, and 15–16% and 19–20% higher Healthy Eating Index (HEI)-2015 scores, respectively, compared with the typical US pattern [118]. Similarly, a cluster analysis identifying bean dietary patterns found that adults consuming beans (ranging from ~1.7 to ~2 servings daily) had significantly higher diet quality scores (HEI-2015: 55.2–61.2) compared with non-consumers (48.8), along with higher intakes of shortfall nutrients including dietary fibre, iron, magnesium, potassium, folate, choline, and vitamin E [119]. Bean consumption patterns from the same analysis were also associated with lower BMI, decreased body weight, and improved waist circumference. A 2025 nationally representative UK population study found that pulse- and legume-rich diets were associated with higher intakes of fibre, biotin, folate, thiamine, vitamin E, zinc, magnesium, phosphorus, potassium and manganese and with lower intakes of saturated fats, total and free sugars [120]. Pulse and legume consumers also had higher plasma selenium and total carotenoid concentrations, and a single portion (80 g) of legumes was associated with a 3.7-point increase in the EAT-Lancet sustainable diet quality index. One cup of cooked navy beans provides approximately 19 g of dietary fibre, 255 µg of folate, 4.3 mg of iron, 96 mg of magnesium, and 708 mg of potassium [121]. These nutrients are identified as shortfalls of public health concern in Western populations, demonstrating that a single daily serving delivers clinically meaningful micronutrient contributions. For cancer survivors specifically, higher fibre intake during survivorship has direct functional relevance beyond cancer risk reduction: a longitudinal study in 459 CRC survivors followed for 24 months post-treatment found that an intraindividual increase of 10 g/day in dietary fibre over time was significantly associated with better physical functioning (β = 2.3, 95% CI: 0.1–4.4), improved role functioning (β = 5.9, 95% CI 1.5–10.3), and less fatigue (β = −4.1, 95% CI: −7.7 to −0.5), independent of other covariates [122]. Legumes are recognised in the cancer survivorship nutrition literature for their nutrient density. A review on nutrition in cancer survivorship describes legumes, including pulses, as rich sources of plant protein, fibre, and key micronutrients such as zinc, folate, iron, B vitamins, and magnesium, nutrients relevant to recovery and long-term health [123].

However, three important clarifications must accompany this optimistic picture. First, the observed micronutrient improvements in the included trials were achieved under controlled feeding conditions where study meals and snacks were prepared and provided to participants, a setting that does not necessarily reflect what occurs when individuals attempt to self-incorporate beans into habitual free-living diets. A systematic review of dietary interventions for cancer survivors [124] found that while interventions consistently demonstrated improvements in fruit and vegetable intake and dietary fibre, effects were often modest and inconsistent across studies, with self-directed dietary change producing smaller and less reproducible nutrient-level improvements than controlled feeding designs. Second, evidence on gastrointestinal (GI) tolerability remains very limited: only one small feasibility trial reported GI symptom outcomes, describing minimal discomfort and similar symptoms to control meals, whereas other bean interventions in this review focused on adherence and mechanistic biomarkers without systematically assessing GI symptom burden. Third, in the navy bean feasibility trial by Borresen et al. [41], CRC survivors and non-cancer participants showed different intake responses: navy bean powder increased fibre intake among CRC survivors over 4 weeks, whereas non-cancer participants reduced total energy and carbohydrate intake, without similar changes documented in the CRC group. This pattern suggests that the energy-moderating effects of bean-rich foods observed in generally healthy adults may not translate directly to CRC survivors, underscoring the need for larger, stratified trials to clarify satiety and energy intake responses in post-treatment populations.

### 4.4. Heterogeneity of Included Studies

A critical but underappreciated source of heterogeneity in the current evidence base is the form in which pulses are consumed prior to ingestion. Processing methods, including soaking, boiling, pressure cooking, fermentation, germination, and milling into powder, exert substantial and sometimes opposing effects on the bioactive compounds most plausibly linked to cancer risk reduction. Open (conventional) cooking of pulses has been shown to alter total phenolic content at rates ranging from −8.38% to 9.29% and flavonoid content between −42.8% and 58.5%, while pressure cooking produces a more consistent increase in total flavonoids of 22% to 40.3% [125]. Soaking and hydrothermal processing significantly reduce tannin concentrations by up to 99% in some legumes, thereby improving the bioavailability of protein and minerals, though their effect on phytates is comparatively modest [126]. Fermentation further improves nutritional quality by reducing anti-nutritional factors and enhancing mineral bioavailability, and fermented legume products have demonstrated greater biological activity in experimental models compared with their non-fermented counterparts [127]. However, the relationship between preparation method and bioactive content is not uniformly favourable. Soaking and boiling in particular can leach water-soluble phenolics and antioxidants into cooking water, and fresh green beans, as consumed in the South African oesophageal cancer study [44], have been found to retain higher antioxidant concentrations compared with their canned or boiled counterparts [128]. This suggests that the protective signal observed for frequent green legume intake in women may partly reflect the higher polyphenol bioaccessibility of fresh forms rather than legume consumption per se. Furthermore, roasting common food beans (*Phaseolus vulgaris*) has been shown to increase certain phenolic compounds and antioxidant capacity compared with unprocessed forms, though the effect varies by variety and roasting method [129], indicating that the fresh-versus-processed distinction does not uniformly favour fresh forms across all preparation types.

Comparison across the included studies is further complicated by the use of fundamentally different pulse forms that were never explicitly harmonised. The five mechanistic RCTs [35,36,37,40,41] all used a standardised navy bean powder (Vege Full™) at 35 g/day, while the BE GONE crossover trial [39] used pressure-cooked canned navy beans at approximately two servings per day. These two forms differ substantially in their starch digestibility: starch hydrolysis in whole cooked navy beans reaches approximately 60%, compared with 85–90% in milled bean flour after equivalent in vitro gastrointestinal digestion, because the intact cotyledon cell structure of whole beans physically resists enzymatic access to starch granules [104,130]. This means that navy bean powder may deliver more rapidly digestible starch to the small intestine, while whole cooked beans may deliver more fermentable substrate to the colon, the site of CRC carcinogenesis, though the mechanistic implications of this difference for CRC risk are unclear. Steam-heating and conventional cooking of legumes generates 3–5 times more resistant starch than raw pulses through the retrogradation of amylose chains [131,132]. Meanwhile, conventionally boiled common beans consistently contain higher retrograded resistant starch than pressure-cooked counterparts. Pressure cooking can reduce resistant starch by up to 15% in legumes such as pea, attributed to changes in cell wall integrity that increase enzymatic accessibility to starch granules [133,134]. Resistant starch fermentation in the colon produces short-chain fatty acids, particularly butyrate, which reduce colonic luminal pH and lower concentrations of soluble deoxycholic acid, both of which are inversely associated with CRC risk in systematic meta-analyses [135,136,137]. Furthermore, an intact bean cell structure modulates the spatial delivery of starch to distal colon regions: using a SHIME^®^ fermentation model, higher cellular integrity in red kidney bean cotyledon cells resulted in greater starch delivery to the distal colon and significantly increased *Bifidobacterium* proliferation compared with disrupted cell preparations [83]. This mechanistic distinction may help explain why the BE GONE trial [39], using whole pressure-cooked canned beans, produced significant microbiome shifts including increased *Faecalibacterium* and *Bifidobacterium*, despite the fact that stool SCFAs did not change significantly in the navy bean powder trials [35].

In the observational studies included in this review, preparation form was uniformly absent from exposure definitions. Food frequency questionnaires (FFQs) used by the US cohort [43], the Spanish cohort [45] and the Jordanian case–control studies [32,33] capture the frequency and sometimes the portion size of listed foods, but do not collect detailed information about food preparation, specific forms consumed, or cooking methods [138]. In this review, several legume exposures were pulse-dominant but not restricted to clearly dried forms, with items such as peas and lima beans of unspecified form in the Jordanian CRC case–control studies [32,33], the Iranian and US breast cancer cohorts [42,43], and the Chinese cancer mortality cohort [46], so these were treated as pulse-dominant legume exposures. Participants classified in the same legume intake category in such studies may have consumed pulses in forms with substantially different bioactive profiles, ranging from freshly boiled dried lentils to commercially canned beans to bean-based processed foods. This represents a form of non-differential misclassification that typically attenuates true associations toward the null [139,140]. This mechanism is consistent with the observed pattern in the Chinese cohort [46], where legume intake was inversely associated with cancer mortality in minimally adjusted models but lost significance after full adjustment for socioeconomic and lifestyle factors. Individuals with more favourable socioeconomic profiles may also be more likely to consume pulses in nutritionally superior preparation forms (e.g., home-cooked dried beans rather than sodium-laden canned varieties), creating residual confounding factors by preparation quality that cannot be separated from socioeconomic status in FFQ-based studies. It should be noted, however, that not all evidence supports preparation form as a critical modifying variable. In a study of common beans (*Phaseolus vulgaris*), antioxidant activity and phenolic content after cooking and in vitro GI digestion were not directly related to anti-proliferative activity in cancer cell lines [141], suggesting that the chemoprotective effects of pulses may operate through pathways such as fibre fermentation, microbiome modulation, and dietary displacement of red meat, which are less sensitive to preparation-induced changes in phenolic content.

Collectively, these considerations suggest that preparation method constitutes an insufficiently characterised dimension of pulse exposure that may contribute meaningfully to the heterogeneity observed across studies in this review. Future observational studies should incorporate preparation-specific exposure categories in dietary assessment instruments, distinguishing, at minimum, between fresh, green, dried-and-cooked, canned, fermented, and powdered forms. Future intervention trials should systematically compare whole-bean versus flour or powder forms to disentangle the effects of cell structure integrity, resistant starch content, and phenolic bioaccessibility on mechanistic outcomes. Until such data are available, pooling across studies that used fundamentally non-equivalent pulse exposures should be approached with caution, and the null findings reported in several included cohorts may plausibly reflect preparation-related exposure misclassification rather than a true absence of biological effect. Evidence from this scoping review shows several null and attenuated associations that temper the overall conclusions about pulses and cancer risk. For CRC, one Jordanian case–control study [33] including 167 CRC cases and 240 controls found no significant association between weekly lentil consumption and CRC risk, while another study of 220 CRC cases and 281 controls reported only a non-significant protective trend for higher legume intake, particularly lentils and peas [32]. For breast cancer, a 20-year prospective cohort of 88,803 predominantly premenopausal women (2830 incident breast cancer cases) observed no significant direct association between legume intake and breast cancer when legumes were considered independently, even though substituting one daily serving of total red meat with one serving of legumes was associated with a 15% lower overall risk of breast cancer and a 19% lower risk among premenopausal women [43]. For mortality, a Spanish cohort of 66,933 adults [45] reported no significant associations between pulse intake categories (<1, 1–2, or >3 times per week) and cancer mortality after multivariable adjustment, whereas a Chinese cohort of 41,243 adults initially free of cancer and cardiovascular disease found that consuming one to two servings of legumes per week (150 g cooked per serving) was associated with about 29% lower cancer mortality in minimally adjusted models; however, this association was no longer statistically significant after adjustment for additional socioeconomic and lifestyle factors [46]. Oesophageal cancer data are also mixed: in a South African case–control study of 670 oesophageal cancer cases and 1188 controls, women consuming green legumes (peas and green beans) 5–7 days per week had approximately 54% lower odds of oesophageal cancer than never-consumers, while no significant associations were observed in men, and dry bean intake showed no significant association with risk in either sex [44].

These findings highlight considerable heterogeneity and context dependence in pulse–cancer relationships. Protective associations appear stronger and more consistent for some outcomes and subgroups than others: for example, in the Iranian breast cancer case–control study of 350 cases and 700 controls [42], women in the highest tertile of legume intake had 46% lower odds of breast cancer overall, with significant inverse associations among postmenopausal women and normal-weight women, but no significant associations among premenopausal or overweight, obese women. Sex-specific effects were also evident for oesophageal cancer, where frequent green legume consumption was protective only in women, and null findings for dry beans may partly reflect exposure misclassification arising from simple intake frequency categories that did not capture portion size [44]. In metabolic and mechanistic trials, daily navy bean powder at 35 g/day for 4 weeks increased gut bacterial richness [35] and significantly altered stool (31 metabolites vs. baseline, 26 vs. controls) [36] and circulating metabolites [37], yet stool SCFAs and predicted Kyoto Encyclopaedia of Genes and Genomes (KEGG) functions did not change significantly [35]. In a three-arm trial of 29 CRC survivors, navy bean powder increased Serum Amyloid A (SAA) and lipopolysaccharide (LPS) while C-reactive protein (CRP) remained unchanged and telomere length did not differ between groups, even though lower SAA across participants was associated with longer telomeres at week four [40]. The BE GONE randomised crossover trial in 55 adults with prior CRC or advanced polyps further showed that favourable microbiome and metabolite shifts induced by approximately two servings per day of canned navy beans regressed within 4 weeks of returning to a bean-free usual diet, indicating that pulse effects are reversible and depend on sustained intake [39]. Pulses, particularly dry beans and lentils, show the most consistent evidence of benefit for CRC, drawing largely on one pooled case–control analysis of legume (pulse-dominant) intake that reported odds ratios as low as 0.68 for ≥2 weekly portions and an estimated 13% lower risk per additional portion [34]. CRC survivor and high-risk trials demonstrate measurable microbiome, metabolite and nutrient changes at feasible bean doses, but these are small, short-term studies from a single research group and have not yet linked biomarkers to clinical endpoints [35,36,37,39,40]. For breast and oesophageal cancer and all-site cancer mortality, associations are heterogeneous, often subgroup-restricted and sensitive to covariate adjustment, so any pulse-related signals should be regarded as tentative rather than definitive. For breast and oesophageal cancer and for all-site cancer mortality, associations are more heterogeneous, sometimes restricted to specific subgroups (e.g., postmenopausal or normal-weight women [42], or women with high green legume intake [44]) or observed only in minimally adjusted models [46]. Mechanistic studies provide biologically plausible pathways, such as via prebiotic shifts in taxa [39], modulation of glutathione, amino acid and lipid pathways [36,37], and enhancement of fibre and micronutrient intake [40,41], by which pulses could influence carcinogenesis and survivorship. Intervention trials show that these dietary changes are generally well tolerated with high adherence [41]. However, the presence of null and context-dependent findings, modest sample sizes in mechanistic trials (typically 16–55 participants), reliance on self-reported dietary data in observational studies and potential residual confounding mean that definitive causal claims cannot yet be made. Pulses can therefore be considered promising components of cancer-protective dietary patterns, especially for CRC, but the current evidence base still warrants cautious interpretation and further high-quality research.

### 4.5. Translational Challenges: From Preclinical Models to Human Dietary Practice

The potential anti-cancer effects reported for pulse extracts and isolated constituents in cell and animal models need to be interpreted with caution when extrapolating to human populations, because the experimental conditions differ from habitual dietary exposure in three key respects. First, the concentrations of bioactive compounds that suppress proliferation or induce apoptosis in vitro are typically one to two orders of magnitude higher than the peak plasma levels achievable after realistic pulse servings. In addition, most in vitro studies do not take into account extensive phase-II metabolism into glucuronidated and sulphated conjugates, which may have weaker antiproliferative activity than the aglycone forms usually tested in cell systems [18,142]. Second, the fraction of these compounds that is actually liberated from the pulse matrix, absorbed, and delivered to target tissues is constrained by low and highly variable bioavailability; polyphenol and saponin bioaccessibility depends on cotyledon cell integrity, particle size, and inter-individual differences in gut microbiota, so total phenolic content in raw beans is a poor proxy for in vivo exposure [143,144]. Third, virtually all human intake occurs in cooked, soaked, canned or otherwise processed forms, and thermal and hydrothermal processing can reduce or redistribute key bioactive classes such as polyphenols, saponins, resistant starch and phytates by roughly 20–80%, with the direction and magnitude of change differing by species and cooking method [145,146,147]. These discrepancies in dose, bioavailability and preparation mean that promising chemopreventive signals from preclinical pulse research should be viewed as hypothesis-generating; future human studies need to use doses that are physiologically realistic, characterise the bioavailability and metabolite profiles of pulse bioactives, and carefully document and standardise preparation methods if they are to close the translational gap between experimental models and real-world dietary practice.

### 4.6. Limitations of the Review Process

Several methodological limitations inherent to this scoping review design should be acknowledged. First, the literature searches were confined to three electronic databases (CENTRAL, Scopus, PubMed) and English-language publications from 2014 to 2025, potentially overlooking relevant grey literature, non-English studies, conference proceedings, or pre-2014 evidence. In addition to the pre-specified database and citation searches, exploratory post hoc web-based and grey literature searches were undertaken to look for additional human studies of pulses and cancer, including other commonly consumed pulse types such as mung beans, common beans and adzuki beans, but these did not identify any eligible human cancer-outcome studies beyond those captured in the main search. The absence of these pulse types in the included studies may reflect both true gaps in the human evidence base and variability in indexing or terminology, and consequently limits the cultural and dietary generalisability of the findings. Second, although no prospective protocol was registered, methods adhered to the PRISMA-ScR and JBI guidance with predefined PCC criteria, which may introduce risk of post hoc modifications despite rigorous reviewer processes. Third, the omission of formal critical appraisal of individual study quality, which is appropriate for scoping reviews mapping evidence breadth, precludes strength-of-evidence grading. Finally, narrative synthesis across heterogeneous designs (RCTs, case–control, cohorts) and pulse preparations limits quantitative meta-analysis and effect size comparisons. These limitations delineate opportunities for targeted systematic reviews with expanded sources and quality assessment.

## 5. Conclusions

In summary, this scoping review indicates that pulses are promising but incompletely characterised components of cancer-protective dietary patterns. Across case–control and pooled observational analyses, higher pulse intake is compatible with modestly lower CRC risk, whereas findings for breast, oesophageal and all-cancer mortality are mixed, often confined to specific subgroups or attenuated after fuller adjustment for lifestyle and socioeconomic factors. Short-term mechanistic trials in CRC survivors and high-risk adults demonstrate that feasible doses of navy beans can alter gut microbial composition, metabolites, fibre and micronutrient intake, yet these changes have not been linked to reductions in clinical endpoints such as adenoma recurrence or cancer progression. Moreover, five of the navy bean interventions were delivered by a single research group using near-identical protocols in overlapping survivor populations, which tempers the apparent consistency of mechanistic evidence and underscores the need for independent replication. Taken together, these data support a cautious interpretation: pulses are nutrient-dense, low-glycaemic foods that can potentially help implement the plant-rich dietary patterns recommended for cancer prevention and survivorship, but current human evidence is insufficient to infer causal, pulse-specific effects on cancer outcomes. Important gaps include limited data outside CRC, under-representation of high pulse-consuming regions, and substantial exposure misclassification in observational cohorts, particularly around portion size and preparation form. In several cohorts, null or weak associations may plausibly reflect these measurement limitations rather than an absence of biological effect. Future work should move beyond short feasibility trials and generic “legume” categories by prioritising long-duration pulse-specific interventions (≥1–2 years) with clinical endpoints, incorporating detailed characterisation of preparation methods (e.g., whole beans versus flours, canned versus home-cooked, fermented products), and embedding objective biomarkers of intake and response within multi-omics frameworks. Well-designed cohort studies and trials in diverse dietary cultures, alongside the integration of pulses into survivorship care pathways and dietary pattern research, are needed before pulse-focused recommendations for cancer risk reduction and their potential role in survivorship care can be formulated with confidence. Pulses can therefore be considered promising components of cancer-protective dietary patterns, especially for CRC, but the current evidence base still warrants cautious interpretation and further high-quality research. In addition, the substantial inter-species variation in nutrient composition, protein digestibility, fibre content, and antioxidant capacity documented across pulse types means that aggregating all pulses into a single exposure may obscure species-specific effects that are biologically meaningful. Recognising and explicitly modelling this heterogeneity will be essential if future observational and intervention studies are to disentangle whether particular pulse species, rather than pulses as a broad class, drive the cancer-related associations observed to date.

## Figures and Tables

**Table 1:** Search strategy.

Database	Search Strategy
Cochrane Central Register of Controlled Trials (CENTRAL)	“Adult” OR “Young Adult” OR “Middle Aged” OR “Man” OR “Woman” OR “Men” OR “Women” OR “Patient” OR “Person” OR “Human” OR “Individual” in Title Abstract Keyword AND “dry edible seeds” OR “dry beans” OR “kidney beans” OR “black beans” OR “cannellini beans” OR “navy beans” OR “pinto beans” OR chickpeas OR “dry peas” OR “split peas” OR “black-eyed peas” OR lentils in Title Abstract Keyword AND “cancer” OR “neoplasm” in Title Abstract Keyword
Scopus	(TITLE-ABS-KEY (“Adult” OR “Young Adult” OR “Middle Aged” OR “Man” OR “Woman” OR “Men” OR “Women” OR “Patient” OR “Person” OR “Human” OR “Individual”) AND TITLE-ABS-KEY (“dry edible seeds” OR “dry beans” OR “kidney beans” OR “black beans” OR “cannellini beans” OR “navy beans” OR “pinto beans” OR chickpeas OR “dry peas” OR “split peas” OR “black-eyed peas” OR lentils) AND TITLE-ABS-KEY (“cancer” OR “neoplasm”)) AND PUBYEAR > 2013 AND (LIMIT-TO (DOCTYPE, “ar”)) AND (LIMIT-TO (LANGUAGE, “English”)) AND (LIMIT-TO (EXACTKEYWORD, “Human”))
PubMed	((“Adult”[Title/Abstract] OR “Young Adult”[Title/Abstract] OR “Middle Aged”[Title/Abstract] OR “Man”[Title/Abstract] OR “Woman”[Title/Abstract] OR “Men”[Title/Abstract] OR “Women”[Title/Abstract] OR “Patient”[Title/Abstract] OR “Person”[Title/Abstract] OR “Human”[Title/Abstract] OR “Individual”[Title/Abstract]) AND (“dry edible seeds”[Title/Abstract] OR “dry beans”[Title/Abstract] OR “kidney beans”[Title/Abstract] OR “black beans”[Title/Abstract] OR “cannellini beans”[Title/Abstract] OR “navy beans”[Title/Abstract] OR “pinto beans”[Title/Abstract] OR chickpeas[Title/Abstract] OR “dry peas”[Title/Abstract] OR “split peas”[Title/Abstract] OR “black-eyed peas”[Title/Abstract] OR lentils[Title/Abstract])) AND (“cancer”[Title/Abstract] OR “neoplasm”[Title/Abstract])

**Table 2:** Tabulated summary of articles included in this review.

Reference, Country (Year)	Design and Population	Pulse Exposure	Pulse Preparation	Cancer Site and Outcome	Main Findings
**Colorectal cancer—incidence**					
[32], Jordan (2016)	Case–control; 220 CRC cases, 281 controls	Lentils, peas; higher vs. lower intake	Not specified	CRC—incidence	^†^ Higher intake of legumes, particularly lentils and peas suggested a protective trend against CRC, but associations were not statistically significant.
[33], Jordan (2015)	Case–control; 167 CRC cases, 240 controls	Lentils; weekly consumption	Not specified	CRC—incidence	Weekly lentil consumption showed no significant association with CRC risk.
[34], Italy/Switzerland (2024)	Pooled case–control; 10,482 cancer cases across 10 sites	Dry beans, chickpeas, dry peas, lentils; <1, 1, or ≥2 portions/week	Not specified	CRC—incidence	^‡^ Legume intake was inversely associated with CRC risk (OR 0.79 for ≥1 portion/week; OR 0.68 for ≥2 portions/week), with ≈13% lower risk per additional weekly portion; benefits may partly reflect replacement of processed meats/high-GI foods.
**Colorectal cancer—survivors/high-risk (mechanistic and feasibility)**					
[35], USA (2017)	RCT, 4 weeks; overweight/obese CRC survivors (*n* = 29)	Navy bean powder 35 g/day	Cooked navy beans processed into powder and incorporated into study meals/snacks	CRC—survivors (microbiome, SCFAs, fibre)	Navy bean powder increased gut bacterial richness at 28 days and total dietary fibre intake but did not change overall diversity, community structure, phylum-level composition, predicted KEGG functions, or stool SCFAs.
[36], USA (2019)	RCT, 4 weeks; overweight/obese CRC survivors (*n* = 18)	Navy bean powder 35 g/day	Precooked navy beans processed into powder and incorporated into meals/snacks	CRC—survivors (stool metabolome)	Navy bean intake altered 31 stool metabolites vs. baseline and 26 vs. controls, with 237 potential bean-derived metabolites and pathway changes in glutathione, amino acid, and lipid metabolism, plus increased antitumor phytochemicals.
[37], USA (2021)	RCT, 4 weeks; overweight/obese CRC survivors (*n* = 20)	Navy bean powder 35 g/day	Precooked whole navy beans processed into powder and added to one meal and one snack/day	CRC—survivors (plasma and urine metabolome)	Navy bean consumption increased plasma 2,3-dihydroxy-2-methylbutyrate, pipecolate, and S-methylcysteine and urinary N2,N5-diacetylornithine and 4-hydroxyhippurate; several metabolites were navy bean-derived and targeted amino acid and lipid pathways implicated in reducing CRC recurrence.
[38], USA (2015)	Controlled feeding + observational; 46 men in crossover trial; 212 polyp-free PPT participants	Cooked pinto, navy, kidney, lima, and black beans; high-bean diet 250 g/day	Cooked beans; PPT also included common beans such as baked, kidney, pinto, lima, and navy beans	CRC—risk reduction context (serum biomarkers)	Dry bean intake markedly increased serum pipecolic acid and S-methyl-cysteine, plus N-acetylornithine, trigonelline, and indole propionate; 80 serum metabolites changed after the high-bean diet; PA and SMC strongly tracked long-term bean intake.
[39], USA (2023)	Prospective randomised crossover; high-risk CRC patients and CRC survivors (*n* = 55)	Organic navy beans (2 servings)	Pressure-cooked, canned organic navy beans added to the usual diet	CRC—high-risk/survivors (microbiome, metabolome, inflammatory proteome)	Bean intake increased alpha diversity and shifted multiple genera, increased circulating pipecolic acid, SAM, and trigonelline, decreased an indole derivative, and reduced several inflammatory/immune markers; these changes reversed within 4 weeks after beans were stopped.
[40], USA (2016)	RCT, 4 weeks; CRC survivors in 3-arm trial	Navy bean powder 35 g/day	Whole navy beans washed, soaked, cooked, ground, and dehydrated to powder; added to frozen meals/snacks	CRC—survivors (micronutrients, inflammation, telomeres)	Navy bean powder increased intake of iron, magnesium, zinc, folate, and vitamin B6; SAA and LPS increased but SAA remained within normal range; CRP did not differ; telomere length did not change, but lower SAA at week 4 was associated with longer telomeres across participants.
[41], USA (2014)	Placebo-controlled RCT, 4 weeks; 7 non-cancer adults and 9 CRC survivors	Navy bean powder 35 g/day	Whole navy beans washed, soaked, cooked, ground, and dehydrated; powder split between one meal and one snack/day (Vege Full™)	CRC—survivors and non-cancer (diet quality, tolerability)	In CRC survivors, navy bean powder increased fibre intake from week 2 to week 4; in non-cancer participants, it reduced total calories and carbohydrate intake; meals/snacks provided more calcium, potassium, zinc, and folate; GI discomfort was minimal and navy bean powder was practical to use.
**Breast cancer**					
[42], Iran (2021)	Population-based case–control; 350 breast cancer cases, 700 controls	Lentils, chickpeas, peas, pinto beans, red beans; top vs. bottom tertile	Assessed as part of cooked or canned mixed dishes	Breast—incidence (odds)	^†^ Higher legume intake was associated with 46% lower odds of breast cancer overall; inverse associations were significant in postmenopausal and normal-weight women, but not in premenopausal or overweight/obese women.
[43], USA (2014)	Prospective cohort; 88,803 premenopausal women followed 20 years	Beans, lentils, peas, lima beans	Not specified	Breast—incidence	^†^ Legume intake was not significantly associated with breast cancer risk; however, replacing one serving/day of red meat with legumes was associated with 15% lower breast cancer risk overall and 19% lower risk among premenopausal women.
**Other cancer sites and cancer mortality**					
[44], South Africa (2014)	Hospital-based case–control; 670 oesophageal cancer cases, 1188 controls	Dry beans; frequency of intake	Not specified	Oesophageal—incidence (odds)	Dry beans showed no significant association in either sex.
[45], Spain (2025)	Prospective study; 66,933 adults from 3 national surveys	Pulses (not specified); <1, 1–2, >3 times/week	Not specified	All cancers—mortality	After adjustment for health and lifestyle factors, pulse intake was not significantly associated with cancer mortality.
[46], China (2021)	Prospective cohort; 41,243 adults, cancer- and CVD-free at baseline	Beans, lentils, chickpeas, black beans, peas, black-eyed peas; servings/week	Not specified	All cancers—incidence and mortality	^†^ In minimally adjusted models, legume intake was inversely associated with cancer incidence and mortality, with about 29% lower cancer mortality for 1–2 servings/week; these associations were attenuated after full adjustment.

^†^ In these studies, legume exposures were FAO-defined pulses; nonetheless, because the form of some items was not detailed, consumption in non-dried forms cannot be ruled out. ^‡^ In these studies, legume exposures were FAO-defined pulses. Abbreviations: CRC—Colorectal Cancer; OR—Odds Ratio; GI—Glycaemic Index; KEGG—Kyoto Encyclopaedia of Genes and Genomes; SCFA—Short-Chain Fatty Acid; PA—pipecolic acid; SMC—S-methyl-cysteine; SAA—Serum Amyloid A; SAM—S-Adenosylmethionine; LPS—Lipopolysaccharide; CRP—C-Reactive Protein; RCT—Randomised Controlled Trial; PPT—Polyp Prevention Trial; CVD—cardiovascular disease.

## Data Availability

No new data were created or analysed in this study. Data sharing is not applicable to this article.

## Supplementary Materials

The following supporting information can be downloaded at: https://www.mdpi.com/article/10.3390/nu18132064/s1, Table S1: Preferred Reporting Items for Systematic reviews and Meta-Analyses extension for Scoping Reviews (PRISMA-ScR) Checklist.

## Abbreviations

The following abbreviations are used in this manuscript:

BE GONE	Beans to Enrich the Gut microbiome vs. Obesity’s Negative Effects
BMI	body mass index
CENTRAL	Cochrane Central Register of Controlled Trials
CI	confidence interval
CRC	colorectal cancer
ESCC	esophageal squamous cell carcinoma
FAO	Food and Agriculture Organisation
FFQ	food frequency questionnaire
GI	gastrointestinal
GSH	reduced glutathione
HEI	healthy eating index
HR	hazard ratio
IGF-1	insulin-like growth factor-1
JBI	Joanna Briggs Institute
KEGG	Kyoto Encyclopaedia of Genes and Genomes
LPS	lipopolysaccharide
NHANES	National Health and Nutrition Examination Survey
OR	odds ratio
PCC	population, concept, and context
PRSIMA-ScR	Preferred Reporting Items for Systematic reviews and Meta-Analyses extension for Scoping Reviews
RCT	randomised clinical trials
RR	relative risk
SAA	serum amyloid A
SAM	S-adenosyl-l-methionine
SCFA	short-chain fatty acids
TGFB1	transforming growth factor Beta 1
